# Palmitate-induced ER stress and inhibition of protein synthesis in cultured myotubes does not require Toll-like receptor 4

**DOI:** 10.1371/journal.pone.0191313

**Published:** 2018-01-12

**Authors:** Ben D. Perry, Jill A. Rahnert, Yang Xie, Bin Zheng, Myra E. Woodworth-Hobbs, S. Russ Price

**Affiliations:** 1 Department of Medicine, Renal Division, Emory University, Atlanta, GA, United States of America; 2 Center for the Study of Human Health, Emory College of Arts and Sciences, Emory University, Atlanta, GA, United States of America; 3 Atlanta VA Medical Center, Decatur, GA, United States of America; 4 Department of Biochemistry and Molecular Biology, Brody School of Medicine, East Carolina University, Greenville, NC, United States of America; Duke University School of Medicine, UNITED STATES

## Abstract

Saturated fatty acids, such as palmitate, are elevated in metabolically dysfunctional conditions like type 2 diabetes mellitus. Palmitate has been shown to impair insulin sensitivity and suppress protein synthesis while upregulating proteolytic systems in skeletal muscle. Increased sarco/endoplasmic reticulum (ER) stress and subsequent activation of the unfolded protein response may contribute to the palmitate-induced impairment of muscle protein synthesis. In some cell types, ER stress occurs through activation of the Toll-like receptor 4 (TLR4). Given the link between ER stress and suppression of protein synthesis, we investigated whether palmitate induces markers of ER stress and protein synthesis by activating TLR4 in cultured mouse C2C12 myotubes. Myotubes were treated with vehicle, a TLR4-specific ligand (lipopolysaccharides), palmitate, or a combination of palmitate plus a TLR4-specific inhibitor (TAK-242). Inflammatory indicators of TLR4 activation (IL-6 and TNFα) and markers of ER stress were measured, and protein synthesis was assessed using puromycin incorporation. Palmitate substantially increased the levels of IL-6, TNF-α, CHOP, XBP1s, and ATF 4 mRNAs and augmented the levels of CHOP, XBP1s, phospho-PERK and phospho-eIF2α proteins. The TLR4 antagonist attenuated both acute palmitate and LPS-induced increases in IL-6 and TNFα, but did not reduce ER stress signaling with either 6 h or 24 h palmitate treatment. Similarly, treating myotubes with palmitate for 6 h caused a 43% decline in protein synthesis consistent with an increase in phospho-eIF2α, and the TLR4 antagonist did not alter these responses. These results suggest that palmitate does not induce ER stress through TLR4 in muscle, and that palmitate impairs protein synthesis in skeletal muscle in part by induction of ER stress.

## Introduction

Skeletal muscle atrophy reduces muscle function, quality of life, functional independence, and is a consequence in many diseases and conditions including cancer, heart failure, sepsis, chronic kidney disease and type 2 diabetes mellitus (T2DM) [1, 2]. In T2DM, skeletal muscle becomes metabolically dysregulated through a combination of insulin resistance, inflammation and lipotoxicity, all of which may contribute to the development of muscle atrophy through a combination of impaired protein synthesis and accelerated proteolysis via autophagy, the caspases, and the ubiquitin proteasome pathway [3]. Patients with T2DM also have dyslipidemia and increased lipid accumulation in skeletal muscle which may contribute to insulin resistance, a condition linked to muscle wasting [4–7]. In cultured myotubes, exogenous saturated fatty acids such as palmitate (PA) upregulate pro-inflammatory signaling, impair insulin signaling, reduce myotube size and increase protein degradation, indicating the saturated fatty acids directly affect muscle fiber metabolism [8–11].

The status of the endoplasmic reticulum (sarcoplasmic reticulum in muscle) is a significant determinant of protein homeostasis in muscle cells. Accumulation of unfolded proteins and other physiological stresses produce ER stress which initiates the unfolded protein response (UPR). The UPR is a vital cell survival signaling pathway that acts to both decrease overall protein synthesis and increase the activities of several proteolytic systems [12, 13]. The UPR consists of three signaling branches which are initiated by signals such as the dissociation of BiP (GRP78) from the intracellular receptor domains of the ER. These signals activate combinations of the three stress sensors, protein kinase RNA-like endoplasmic reticulum kinase (PERK), activation transcription factor 6 (ATF6) and inositol-requiring enzyme 1 α (IRE 1α). PERK-initiated signaling is particularly relevant to protein homeostasis due to the considerable range of UPR-related genes it targets [14] and its ability to modulate several aspects of protein metabolism [15, 16]. Activation of PERK directly impacts protein synthesis via phosphorylation of eukaryotic inducible factor 2 α (eIF2α), which inhibits the eIF2•GTP•Met-tRNAi protein translation complex. Downstream of PERK and eIF2α, activation transcription factor 4 (ATF4) and transcription factor C/EBP homologous protein (CHOP) activate autophagy signaling and induce caspase 3, likely via caspase-12 [17]. Indeed, activation of ATF4 induces muscle atrophy in mice and decreases protein synthesis [18, 19], while activation of caspase-3 via CHOP and other sources contributes to the accelerated rate of proteolysis through cleavage of actin and other cellular proteins as well as activation of some subunits of the proteasome in skeletal muscle, but does necessarily induce cell death [20, 21]. The IRE1α- and ATF6-initiated branches of the UPR converge to increase transcription of the spliced variant of X-box binding protein 1 (XBP1s), which is a transcription factor that induces BiP expression, particularly when combined with ATF6 [22–24]. XBP1s also works in conjunction with ATF6 and ATF4 to maximally induce transcription of CHOP [25, 26].

Our lab has previously reported that PA acts directly on C2C12 myotubes to increase the rate of protein degradation and induce caspase-3 [8, 13]. Concomitant with this response, nuclear translocation of the atrophy-related transcription factor, forkhead box O 3a (FoxO3a), was increased as was expression of the atrophy-inducing E3 ubiquitin ligase, atrogin-1 (also known as MAFbx) [8]. In addition, PA increased phosphorylation of eIF2α and reduced protein synthesis in C2C12 myotubes, and a high fat diet induced similar responses in muscle of aged mice [13, 15]. These findings suggest that saturated fats and obesity may act directly on muscle fibers to decrease protein synthesis by inducing the PERK-initiated branch of the UPR.

The mechanisms by which PA induces ER stress in tissues remains elusive. Inflammation and ER stress are intrinsically linked, with ER stress activating pro-inflammatory signaling such as c-Jun N-terminal kinases (JNK) and nuclear factor κ-B (NF-κB) through multiple UPR branches [16, 27]. Furthermore, inflammatory pathways may also induce ER stress. In lung, immune, and liver cells, lipopolysaccharide (LPS) induces ER stress and inflammatory cytokine production [28–30]. These responses to LPS are primarily mediated by activation of toll-like receptor 4 (TLR4) which induces a variety of pro-inflammatory proteins including NF-κB and AP-1 [31]. Although it remains unclear if TLR4 induces ER stress in skeletal muscle, there is evidence suggesting that activation of TLR4 by LPS is affiliated with muscle atrophy. C2C12 myotubes treated with LPS had reduced myotube diameter and increased activation of the ubiquitin proteasome and autophagy [32]. ER stress is linked to ubiquitin proteasome and autophagy signaling, and can reduce protein synthesis [12]. In addition to LPS, saturated fatty acids may also activate TLR4 in muscle [33, 34], suggesting that PA-induced atrophy may be in part due to activation of this receptor and the resulting ER stress.TLR4 is inhibited by TAK-242, a highly specific pharmaceutical inhibitor of TLR4 intracellular signaling, with no inhibitory effects on other TLRs [35, 36]. In myotubes, TAK-242 blocks LPS-induced inflammatory responses [37]. Pierre et al., [38] also demonstrated that global knockout of TLR4 in mice attenuated a high fat diet-induced increase in BiP, XBP1 s and CHOP in skeletal muscle, liver and adipose tissue. However, given that TLR4 expression was ablated in all tissues in that study [38], it is difficult to definitively conclude whether the reduction in muscle ER stress was due to the direct effect of TLR4 knockout in muscle or to the indirect effects of ablating TLR4 in other tissues (e.g., reduced circulating inflammatory signals).

Given the potential overlap between TLR4, ER stress, and protein turnover, we investigated the role of TLR4 on ER stress signaling and protein synthesis in PA-treated C2C12 myotubes. This model eliminates the confounding and indirect effects of PA on other cell types and allows a more direct interrogation of the interaction between TLR4, ER stress, and protein synthesis in muscle cells.

## Methods

### Cell culture

C2C12 myoblasts, derived from mice and obtained from American Type Culture Collection (Manassas, VA, USA) were grown in Dulbecco’s Modified Eagle Medium (DMEM) containing 4.5 g/L glucose and supplemented with 10% fetal bovine serum (FBS; Atlanta Biologicals, Lawrenceville, GA, USA) and antibiotics (1% of final media volume consisting of 100 U/mL penicillin and 100 μg/mL streptomycin; Invitrogen, Carlsbad, CA, USA). At 90–95% confluence, cells were induced to differentiate into myotubes by replacing the growth media with DMEM containing 4.5 g/L glucose plus 2% horse serum (Invitrogen) and antibiotics for 4 days before initiating experimental treatments.

### Experimental treatments

Lipopolysaccharides (LPS; E.Coli 0128:B12, Sigma Aldrich, St. Louis, MO, USA) were diluted in DMEM before being added to the treatment media, which consisted of DMEM plus 2% bovine serum albumin (BSA; Fraction V BSA; Roche, Indianapolis, IN, USA), 2% FBS, and 1% antibiotics at a final concentration of 100 ng/mL. Palmitate (PA) (Sigma Aldrich) was dissolved in ethanol and added to the treatment media at a final concentration of 500 μM. These concentrations of PA and BSA were chosen as 2% BSA and 500 μmol/L free fatty acids are similar to the final molar ratio of human plasma [39], and fasting plasma nonesterified fatty acids are around 600 μmol/L in obese adults with impaired glucose tolerance or diabetes [40]. The TLR4-specific inhibitor TAK-242 (CLI-095, InvivoGen, San Diego, CA, USA) was diluted in DMSO and added to the treatment media at a final concentration of 1 μM [35]. This concentration (1 μM) of TAK-242 specifically inhibits only intracellular TLR4 signaling, and does not affect signaling from other TLRs [35, 36]. Furthermore, 1μM TAK-242 has been previously shown to inhibit LPS induced pro-inflammatory signaling in cultured myotubes [37]. Before the LPS or PA co-treatment with TAK-242, myotubes underwent a 30 minute pre-treatment with TAK-242. Control cells included the vehicle (ethanol and DMSO), and all treatments contained the same final percentage of BSA (2%), ethanol (0.5%) and DMSO (0.1%).

### RNA isolation and qRT-PCR analysis

Total RNA was isolated using TRIzol (Invitrogen, Carlsbad, CA, USA) and reverse transcribed using the Superscript III First-Strand Synthesis kit (Invitrogen) according to the manufacturers’ instructions. Specific mRNAs were measured by quantitative real time PCR in a BioRad iCycler with iQ SYBR Green reagent (BioRad Laboratories, Hercules, CA, USA) and previously published primer sets for ATF4 [41], CHOP, XBP1s [42], MCP-1, TNF-α [43] and IL-6 [44]. 18S rRNA was used as the normalization control. The data were analyzed for fold change (ΔΔCt) using the iCycler software as described previously [45]; treatment values are expressed relative to control. Melt curve analyses were performed to verify the specificity of the reaction.

### Western blot analysis

Whole cell lysates were prepared using buffers specified by the antibody vendors and cleared of cellular debris by centrifugation at 14,000g for 15 min. The concentrations of protein in the lysates were measured using a BioRad DC Protein Assay (BioRad Laboratories, Hercules, CA). Western blot analyses were performed as described previously [46] using nitrocellulose membranes and commercial antibodies to phospho-PERK (T980), PERK, phospho-eIF2α (S51), eIF2α, CHOP, (Cell Signaling Technology, Beverly, MA, USA), spliced XBP1 (clone 9D11A43, Biolegends, San Diego, CA, USA), phospho-p70^S6K^ (Sigma) and p70^S6K^ (Thermo Fisher, Waltham, MA, USA). Equal protein loading and electroblot transfer were verified by staining the membrane post transfer with Ponceau S Red. This method has been routinely used by our lab [45] and has been validated as a reliable measure of protein loading with immunoblotting [47]. It also alleviates the need to validate a control protein each time a treatment or culture condition is modified. Following the chemiluminescence step, densities of detected protein bands were determined using Image J (NIH, Bethesda, MD, USA).

### Protein synthesis assay (SUnSET)

Protein synthesis was measured as previously described using the SUrface SEnsing of Translation (SUnSET) method which measures the incorporation of puromycin into nascent peptide chains [48, 49]. In brief, puromycin dihydrochloride (EMD Millipore, Temecula, CA, USA) was added to the cell treatment media (1 μM final concentration) for 30 min prior to cell lysis with a standard RIPA buffer [45]. Twenty micrograms of protein were separated in a 10% polyacylamide gel under denaturing conditions until the dye-front was ~1.5 cm from the bottom of the gel. Proteins were transferred to nitrocellulose membranes (BioRad) that were subsequently incubated in TBST buffer containing 5% skim milk powder and incubated overnight with a monoclonal antibody to puromycin (clone 12D10, Millipore EMD, diluted 1:5,000) diluted in TBST containing 1% BSA. Membranes were then incubated for 1 h in TBST containing 5% skim milk plus a secondary IgG2a antibody (Jackson ImmunoResearch, West Grove, PA, USA, diluted 1:45,000). The total lane density was analyzed using Image J (NIH).

### Statistical analyses

**Fig 1:**
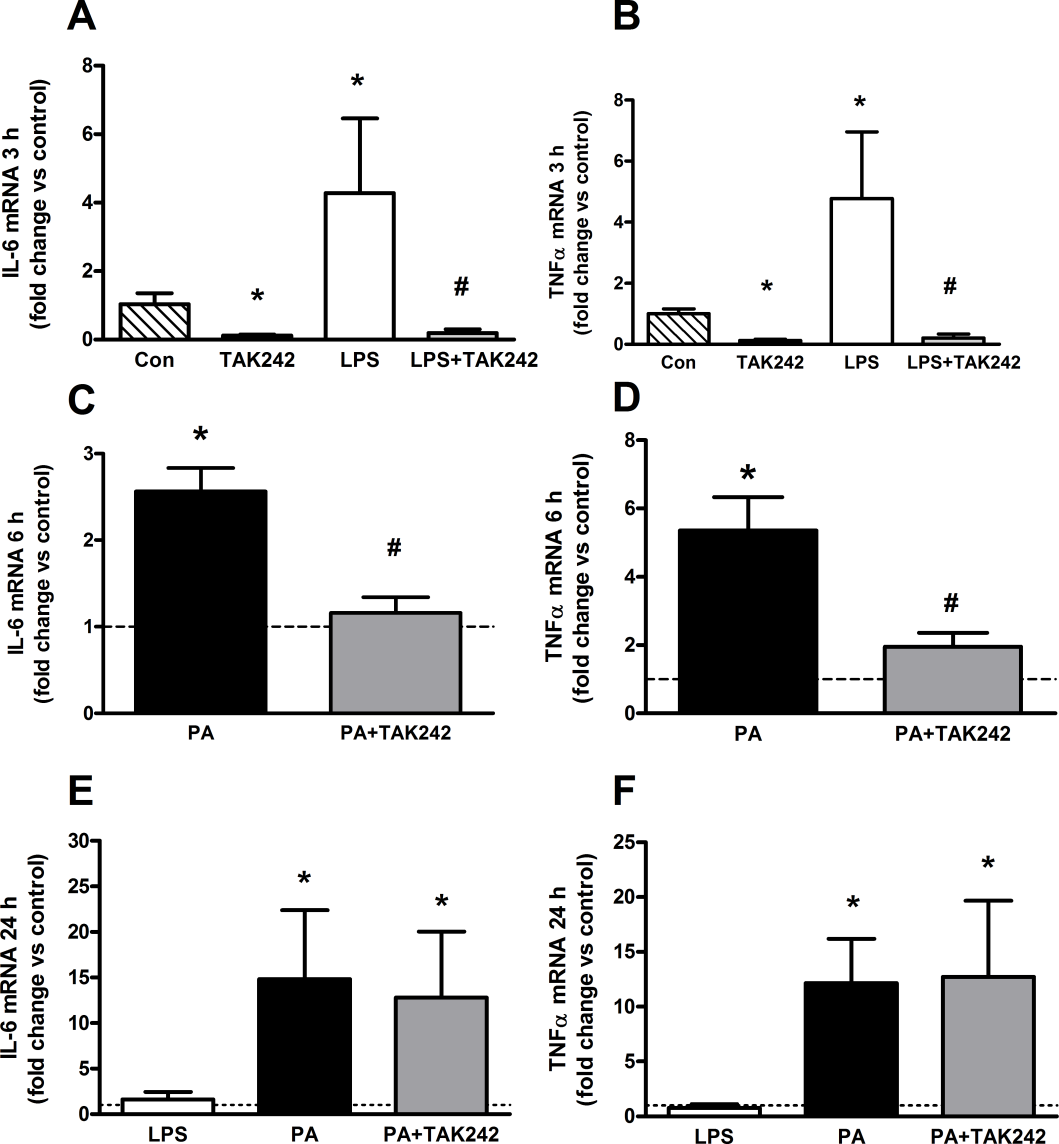
Pharmacological TLR4 inhibition using TAK-242 reversed acute LPS (3 h) and PA (6 h) induced pro-inflammatory mRNA markers IL-6 and TNFα (**A-D**), but did not affect palmitate-induced inflammation in C2C12 myotubes at 24 h **(E-F)**. **A-B**: 3 h of LPS treatment (100 ng/mL) increased IL-6 and TNF-α mRNA which was reversed by TLR4 inhibitor, TAK-242 (1 μM, n = 6). **C-D**: 6 h of palmitate (500 μM) increased both IL-6 and TNFα mRNA expression compared to controls, and was reversed by treatment with TAK-242 (1 μM, n = 3). **E-F**: 24 h of palmitate (500 μM) or palmitate with TAK-242 (1 μM) treatments increased IL-6 and TNF-α mRNA expression relative to controls (n = 4). Values shown are Mean ± SD. * Higher than control (p<0.05), # Lower than LPS treatment (p<0.05).

Data are presented as mean percentage of control ± SD. Differences between treatments are compared by one-way ANOVA with post-hoc analysis using a Fisher’s least significant difference test, except when testing for differences in XBP1s mRNA. In this case, the response to treatment was analyzed using the non-parametric Kruskal-Wallis test. For most experiments, data was calculated as the mean of 3–6 independent experiments, with 2–3 replicates per experiment. The one exception was the experiment which tested the efficacy of TAK-242 in LPS-treated cells (Fig 1A and 1B); in this case; two separate experiments with three replicates per experiment were performed. The statistical sample size is listed in each figure legend. Results are considered statistically significant at p < 0.05. Statistical analyses and figures were generated using Graphpad Prism (GraphPad Software, Inc, La Jolla, CA, USA).

## Results

### TLR4 inhibition attenuates acute LPS-induced mRNA markers of inflammation, but not palmitate-induced inflammation

Both TNFα and IL-6 signaling are commonly reported, reliable indicators of TLR4 activation in the presence of TLR ligands such as LPS [35, 36]. Hence, to demonstrate the efficacy of the TLR4 inhibitor (TAK-242) in our experimental model, we evaluated the effects of LPS and TAK-242 on IL-6 and TNFα mRNA levels in C2C12 myotubes. Consistent with previous studies [37], acute LPS treatment (3 h) caused a 4.3-fold increase in IL-6 and a 4.8-fold increase in TNFα mRNA levels, compared to controls (Fig 1, p<0.05). Both responses were ablated by addition of the TLR4 inhibitor (p<0.05, LPS + TAK242 vs LPS, Fig 1). In addition, IL-6 and TNFα mRNA levels were increased in response to both 6 h (p<0.05, IL-6 mRNA: 1.6-fold increase; TNFα:4.4-fold increase) and 24 h (p<0.05, IL-6 mRNA: 14.8-fold increase; TNFα:15.1-fold increase) PA treatment (500 μM; Fig 1C–1F). TAK-242 inhibited the PA induced increase in IL-6 and TNFα mRNA at the 6 h timepoint (p<0.05), but not 24 h treatment (Fig 1C–1F).

### Palmitate induced ER stress is not prevented by TLR4 inhibition

**Fig 2:**
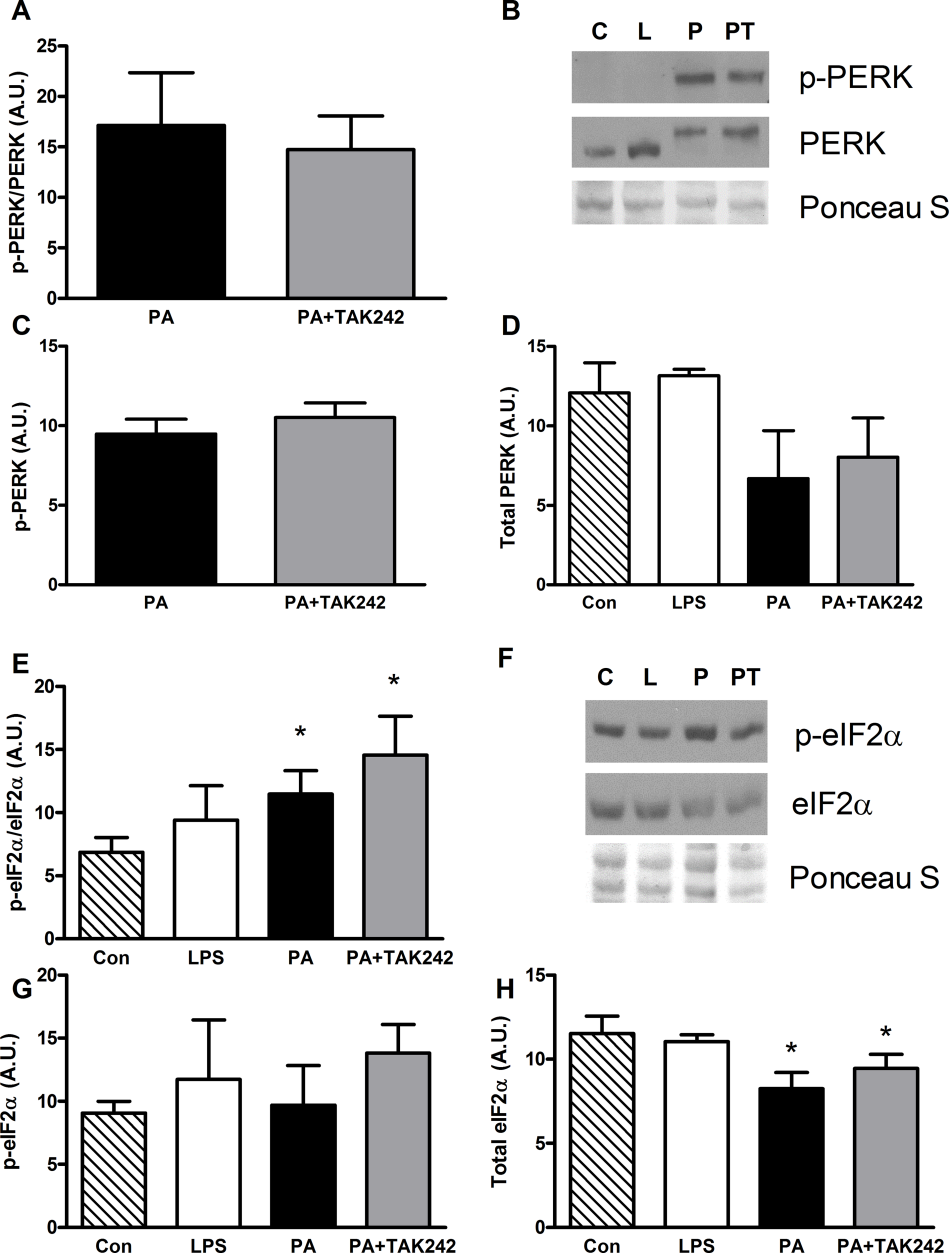
24 h of palmitate treatment increased p-PERK/PERK and p-eIF2α/eIF2α which were not attenuated by TLR4 inhibition in C2C12 myotubes. **A-D**: 24 h of palmitate (500 μM) or palmitate with the TLR inhibitor TAK-242 (1 μM) induced p-PERK (**C**), which was not detectable with control or LPS treatments (n = 4). **E-H**: 24 h of palmitate (500 μM) or palmitate with the TLR inhibitor TAK-242 (1 μM) increased the ratio of p-eIF2α/total eIF2α protein (**E**), and decreased total eIF2α protein (**H**; n = 6). Representative blots for p-PERK/PERK (**B**) and p-eIF2α/eIF2α (**F**) protein; C = control, L = LPS (100 ng/ml), P = palmitate (500 μM), PT = Palmitate (500 μM) and TAK-242 (1 μM). Values shown are Mean ± SD. * Different to control (p<0.05).

Previous work has indicated that PA accelerates proteolysis, and induces both atrophy and ER stress in C2C12 myotubes [8, 9, 15, 50]. The latter finding led us to investigate whether modulation of TLR4 might impact the induction of ER stress and suppression of protein synthesis. In myotubes treated with vehicle alone or LPS to activate TLR4, phosphorylated PERK (p-PERK) was undetectable. In contrast, incubation with PA for 24 h significantly increased the ratio of p-PERK:PERK and co-incubation of PA and TAK242 did not alter the response. (Fig 2A–2D). Consistent with the activation of PERK, PA also increased the ratio of phosphorylated eIF2α (p-eIF2α:eIF2α) (Fig 2E–2H). In this case, the effect of PA was primarily due a reduction in total eIF2α protein. Neither LPS nor TAK-242 affected the PA-induced phosphorylation of eIF2α.

**Fig 3:**
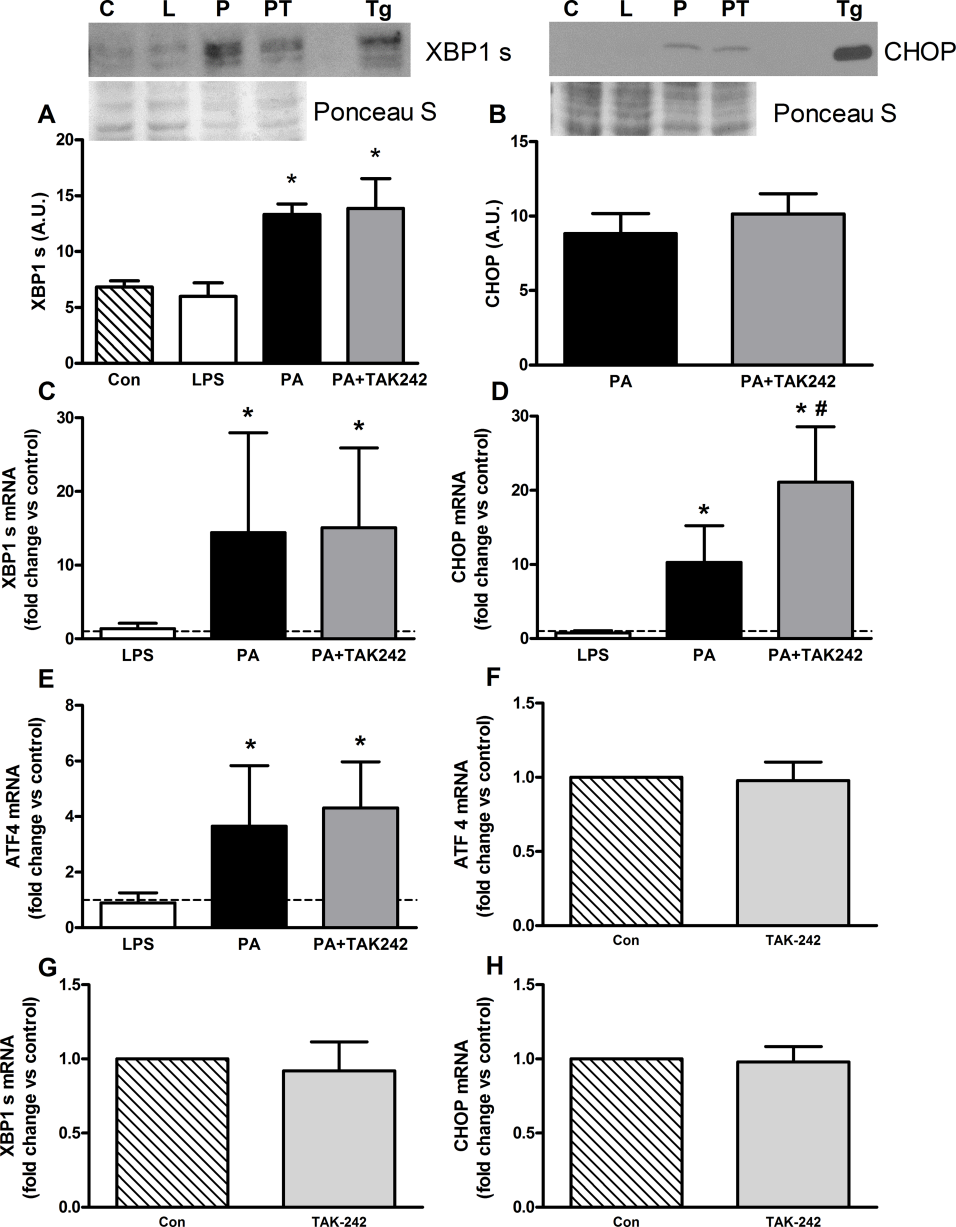
24 h of palmitate treatment increased XBP1 s and CHOP protein expression and were not attenuated by inhibition of TLR4 signaling in C2C12 myotubes. (**A**) 24 h of palmitate (500 μM) or palmitate with the TLR inhibitor TAK-242 (1 μM) increased XBP1 s protein (n = 4) with thapsigargin (Tg) used as a positive control. The fainter, lower band in the representative blot was confirmed as non-specific binding by the manufacturer. (**B**) 24 h of palmitate (500 μM) or palmitate with the TLR inhibitor TAK-242 (1 μM) induced CHOP protein expression (n = 4) with thapsigargin (Tg) used as a positive control. CHOP was not quantifiable in the control or LPS treated samples. (**C**) 24 h of palmitate (500 μM) or palmitate with the TLR inhibitor TAK-242 (1 μM) increased XBP1 s mRNA expression similarly compared to controls (n = 4). (**D**) 24 h of palmitate (500 μM) or palmitate with the TLR inhibitor TAK-242 (1 μM) increased CHOP mRNA expression compared to controls, while combined palmitate and TAK-242 treatment elevated CHOP mRNA to levels higher than palmitate (n = 4). (**E**) 24 h of palmitate (500 μM) or palmitate with TAK-242 (1 μM) increased ATF mRNA expression similarly compared to controls (n = 4). (**F-H**) TAK-242 alone does not independently change XBP1 s, ATF4 or CHOP mRNA levels compared to control (n = 3). Values shown are Mean ± SD. C = control, L = LPS (100 ng/ml), P = palmitate (500 μM), PT = Palmitate (500 μM) and TAK-242 (1 μM). Values shown are mean ± SD. * Higher than control (p<0.05), # Higher than PA (p<0.05).

We also evaluated XBP1s which serves as a marker of the integrated input of ATF6 and IRE1α. XBP1s mRNA and protein were increased by PA (p<0.05, Fig 3A and 3C), but unaffected by co-treatment with TAK-242. Since PERK-eIF2α and XBP1s signaling was activated, we next evaluated the levels of their common downstream effector, CHOP. CHOP protein was induced by PA, but unaffected with the addition of TAK-242 (Fig 3B). Similarly, CHOP mRNA was increased with PA compared to controls (p<0.05). Curiously, the combination of PA plus TAK-242 increased CHOP mRNA more than PA alone (p<0.05, Fig 3D). Consistent with the upstream eIF2α phosphorylation, ATF4 mRNA was increased with PA compared to control (p<0.05, Fig 3E), but not altered with the co-treatment of PA and TAK-242. LPS did not impact ATF4, XBP1s or CHOP mRNA or protein levels, and inhibition of the TLR4 with PA did not affect protein levels of CHOP or XBP1s (Fig 3A–3E). Finally, we confirmed that TAK-242 alone did not alter the basal expression of CHOP, ATF4 or XBP1s mRNAs (Fig 3F).

### Protein synthesis is reduced by palmitate concomitant with increased phosphorylation of eIF2α, and is unaffected by TLR4 inhibition

**Fig 4:**
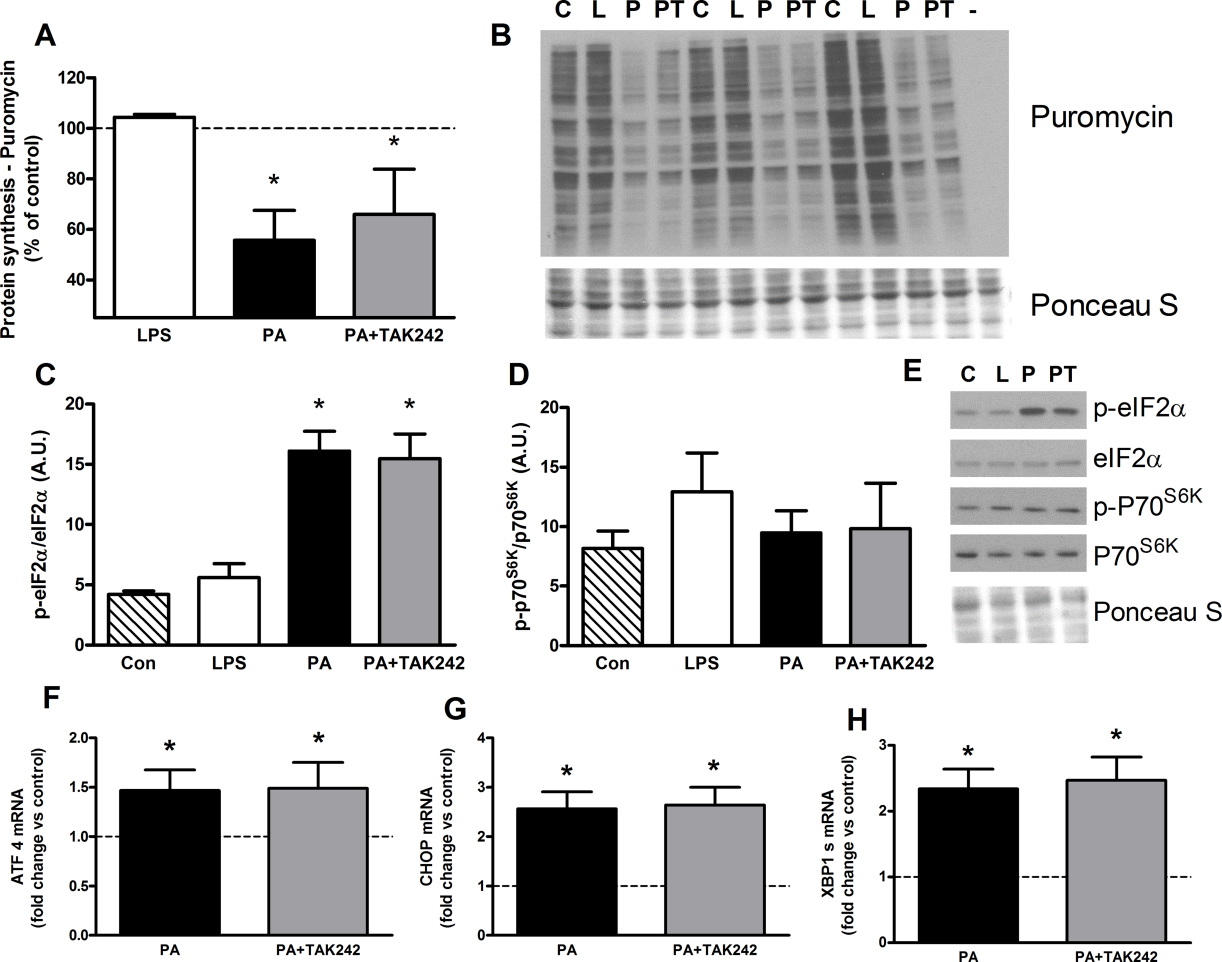
6 h palmitate treatment reduces protein synthesis independent of TLR4 inhibition and concomitant with increased p-eIF2α in C2C12 myotubes. **A**: Protein synthesis, measured using puromycin incorporation, was reduced by both 6 h palmitate treatment (500 μM) and palmitate with TLR inhibitor (TAK-242, 1 μM) co-treatment (n = 3). **B**: Representative puromycin western blot form a single experiment (C = control, L = LPS (100 ng/ml), P = palmitate (500 μM), PT = Palmitate (500 μM) and TAK-242 (1 μM). **C**: 6 h of palmitate (500 μM) or palmitate with the TLR inhibitor TAK-242 (1 μM) increased the ratio of p-eIF2α/total eIF2α protein (n = 3). **D**: No differences were found in p-P70^S6K^/P70^S6K^ following 6 h treatments with LPS, palmitate, or palmitate with TAK-242 co-treatment compared to control (n = 3). **E**: Representative blots for p-eIF2α, eIF2α, p-P70^S6K^ and P70^S6K^ from a single experiment. **F-H**: 6 h palmitate (500μM) increased ATF 4, CHOP and XBP1 s mRNA expression compared to controls, and were not altered with the addition of the TLR4 inhibitor, TAK-242 (1 μM). Dashed line represents control levels (n = 3). Values shown are mean ± SD. * Different to control (p<0.05).

Next we tested whether protein synthesis was suppressed concurrently with activation of ER stress. Protein synthesis was measured using the SUnSET method (46) after cell treatments for 6 h because in earlier studies and pilot data, we found that many of the atrophic responses to PA, such as reduced p-Akt and activation of the ubiquitin proteasome, were most prominent with 4–6 h after PA treatment [8]. Addition of PA to myotubes for 6 hours led to a 43% decrease in protein synthesis which was not changed by the addition of TAK-242 (Fig 4A and 4B). Concomitant with the response to PA, the phosphorylation of eIF2α was increased 3.8 fold. Inhibition of TLR4 did not affect the PA-induced changes in protein synthesis or eIF2α phosphorylation (p<0.05, Fig 4A–4C). We also evaluated the impact of PA on the phosphorylation of the ribosomal protein p70^S6K^, an indicator of mTOR activity. No differences were in the ratio of p-p70^S6K^/p70^S6K^ were found between any of the treatment groups (Fig 4D). Finally, consistent with the findings of eIF2α phosphorylation, TAK-242 did not cause any decrease in ATF4, XBP1 s or CHOP mRNA levels in response to 6 h PA treatment (Fig 4F–4H).

## Discussion

In this study, we investigated the role of TLR4 in PA-induced changes in ER stress and protein synthesis in cultured C2C12 myotubes. Based on current literature, we hypothesized that PA activates TLR4, and that inhibition of TLR4 with TAK-242 in myotubes would attenuate ER stress and the PA-induced suppression of protein synthesis. Our findings confirmed a report by Hussey et al., [37] that TAK-242, a highly-specific TLR4 inhibitor, blocks the acute induction of inflammatory cytokine mRNA transcription by both LPS and saturated fatty acids; however, it did not change the induction of ER stress or the suppression of protein synthesis by PA. Thus, PA-induced ER stress and declines in protein synthesis in skeletal muscle appear to be independent of TLR4 activation *in vitro*.

There are several reports that link TLR4 and ER stress. LPS, a TLR4 ligand, induces expression of inflammatory cytokines as well as ER stress in lung, intestinal, hepatic and immune tissue/cells [28–30, 51]. In contrast, inhibiting TLR4 in our model inhibited initial PA-induced cytokine production, but did not prevent the induction of ER stress. One speculative explanation for this difference is that LPS concentrations such as those used in other studies (e.g., 5–10 mg/kg in mice, 50 ug/mL-10 mg/mL *in vitro*) [28–30, 51] may achieve a higher level of TLR4 activation than possible with the concentrations of PA (300–500 μM) typically used with cultured myotubes [8, 52]. While acute treatment with higher concentrations of PA could potentially be used to test this theory, concentrations of PA higher than ~500 μM for a substantial time period would surpass the total concentrations of nonesterified fatty acids seen in obese and insulin resistant individuals [40]. Considering that PA constitutes only a portion of the total pool of nonesterified fatty acids, it would have been difficult to make any physiologically relevant conclusions in our study if we treated with higher PA concentrations.

Our findings are also in contrast to i*n vivo* findings in mice with a global knockout of TLR4 [38]. In that study, a high-fat diet induced ER stress in the muscles of wild-type mice but not in TLR4-deficient mice. The authors concluded that TLR4 was linked to ER stress in muscle [38]. While this conclusion may be true, the global knockout of TLR4 likely caused other adaptations independent of skeletal muscle, such as adaptations in other organs or tissues like inflammatory cells. Furthermore, unlike control animals, the TLR4 knockout mice did not exhibit any increase in visceral adipose tissue in response to the high-fat diet. The efficacy of TAK-242 in the inhibition of TLR4 is evident in our study. Previous reports have indicated that inhibition of TLR4 with TAK-242 (1μM) consistently inhibits increases in IL-6 and TNFα mRNA levels in multiple cell types [35, 36]. In our study, TAK-242 inhibited acute LPS and PA-induced IL-6 and TNFα mRNA levels, similar to previously reported findings with L6 myotubes [37]. This indicates that TLR4 participates in the initial PA-induced inflammatory cytokine responses in muscle. Importantly, TAK-242 has been demonstrated to have minimal effect on the signaling in other TLRs [35]. Hence, it is highly unlikely that the findings with TAK-242 in our study were due to non-specific signaling in other TLRs, or failure to adequately inhibit TLR4. Unlike with 6 h treatment, TAK-242 did not prevent the induction of IL-6 and TNFα mRNA with 24 h PA treatment. This suggests that while TLR4 is involved in some of the initial induction of pro-inflammatory mRNA expression, longer term expression of IL-6 and TNFα is mediated by TLR4-independent factors. We can only speculate on the mechanisms of this response; one potential explanation for the longer-term induction of IL-6 and TNFα with PA is the increase in mitochondrial damage and oxidative stress [39, 53]. In C2C12 myotubes, PA induced inflammation and ROS production, both of which were both reduced by resveratrol through a SIRT-1 dependent pathway. Furthermore, the induction of prolonged ER stress by PA is also likely to directly contribute to inflammation via TLR4-independent activation of NF-κB [16]; indeed the increase in ATF4, XBP1 s and CHOP mRNA expression were far more substantial with the 24 h PA treatments compared to the acute treatment. Hence, the discovery of TLR4-independent instigators of PA-induced inflammation is an exciting potential area of future research in skeletal muscle.

It is interesting that while the PA-induced upregulation of the UPR was largely unaffected by TLR4 inhibition, there was one notable exception in the increased expression of CHOP mRNA with PA+TAK-242. This effect was not consistent with UPR signaling upstream of CHOP, and perhaps suggests some direct signaling between TLR4 and CHOP exists in muscle. Indeed, macrophages treated with low-dose LPS (1 ng/mL) to induce TLR4 activation had reduced protein expression of p-eIF2α and CHOP in response to PA [54], although what relevance this TLR4 activation has on PA-induced upregulation of the UPR in muscle, and whether this mechanism was involved in our study remains purely speculative. It is noteworthy that despite the increase in CHOP mRNA expression with PA+TAK-242, there was no difference in CHOP protein, suggesting the increased amount of CHOP mRNA expression has little physiological consequence.

Our results indicate that the induction of ER stress, and the resulting activation of the UPR, is likely an important aspect of the cellular response to PA that reduces protein synthesis. In C2C12 myotubes, the reduction in protein synthesis 6 h after PA treatment coincided with increased PERK-eIF2α signaling but no change in p70^S6K^ phosphorylation. Others have reported inconsistent effects of PA on p70^S6K^ phosphorylation in skeletal muscle [52, 55], suggesting that the PA-induced decrease in protein synthesis is more likely the result of increased phosphorylation of eIF2α rather than suppression of mTOR-p70^S6K^ signaling. A report by Tardif et al., [15] found a similar relationship between protein synthesis and the phosphorylation of eIF2α in both older rats fed a high-fat diet for 10 weeks and palmitate-treated myotubes. Hence, ER stress is emerging as an important contributor to the attenuation of protein synthesis in obesity and dyslipidemia. When considering other data indicating that PA upregulates several proteolytic systems in muscle cells, it becomes apparent that PA is a potent regulator of multiple aspects of protein turnover in muscle [8, 15].

There are limitations which should be considered when interpreting our research. Perhaps the most prominent of these limitations is to note that our study was not designed to, and cannot make inferences about the general effect of inflammation or non-TLR4 pro-inflammatory pathways on ER stress and the UPR. The scope of our research was limited to investigating whether TLR4 activation contributed to PA-induced ER stress in skeletal muscle, to which we found that PA-induced ER stress is independent of TLR4 activation. We did not investigate pro-inflammatory pathways in depth, such as NF-κB, and only investigated IL-6 and TNFα as a proxy to assess the effectiveness of TAK-242, and to briefly assess to what extent the IL-6 and TNFα pathways were mediated by TLR4 in response to PA. Our results do not provide any evidence regarding whether TNFα, IL-6, NF-κB or any other important pro-inflammatory signaling axes directly contribute to ER stress, rather we conclude that PA does not induce ER stress in skeletal muscle via activation of TLR4. The contributions of inflammation to the ER stress process, either directly or indirectly, are interesting questions for future research.

In conclusion, our data indicate that TLR4 and its downstream signals are not substantial mediators of PA-induced ER stress in cultured myotubes. The mechanism of ER stress induction by PA can only be speculated from our data, but may be related to an increase in ceramides and other inflammation-linked molecules like ROS that are regulated by NF-κB and JNK [55–58]. It is also possible that PA treatment may produce changes in the membrane phospholipid content of the sarcoplasmic reticulum or mitochondria that are relayed to the ER stress machinery [59]. Further investigations are needed to explore how saturated fatty acids induce ER stress in skeletal muscle, but the findings of our research strongly suggest these factors are independent of TLR4 activation.
